# CRISPR genome editing using computational approaches: A survey

**DOI:** 10.3389/fbinf.2022.1001131

**Published:** 2023-01-11

**Authors:** Roghayyeh Alipanahi, Leila Safari, Alireza Khanteymoori

**Affiliations:** ^1^ Department of Computer Engineering, University of Zanjan, Zanjan, Iran; ^2^ Department of Neurozentrum, Universitätsklinikum Freiburg, Freiburg, Germany

**Keywords:** CRiSPR/Cas, gRNA design, on-target, off-target, computational approach, machine learning

## Abstract

Clustered regularly interspaced short palindromic repeats (CRISPR)-based gene editing has been widely used in various cell types and organisms. To make genome editing with Clustered regularly interspaced short palindromic repeats far more precise and practical, we must concentrate on the design of optimal gRNA and the selection of appropriate Cas enzymes. Numerous computational tools have been created in recent years to help researchers design the best gRNA for Clustered regularly interspaced short palindromic repeats researches. There are two approaches for designing an appropriate gRNA sequence (which targets our desired sites with high precision): experimental and predicting-based approaches. It is essential to reduce off-target sites when designing an optimal gRNA. Here we review both traditional and machine learning-based approaches for designing an appropriate gRNA sequence and predicting off-target sites. In this review, we summarize the key characteristics of all available tools (as far as possible) and compare them together. Machine learning-based tools and web servers are believed to become the most effective and reliable methods for predicting on-target and off-target activities of Clustered regularly interspaced short palindromic repeats in the future. However, these predictions are not so precise now and the performance of these algorithms -especially deep learning one’s-depends on the amount of data used during training phase. So, as more features are discovered and incorporated into these models, predictions become more in line with experimental observations. We must concentrate on the creation of ideal gRNA and the choice of suitable Cas enzymes in order to make genome editing with Clustered regularly interspaced short palindromic repeats far more accurate and feasible.

## 1 Introduction

**FIGURE1:**
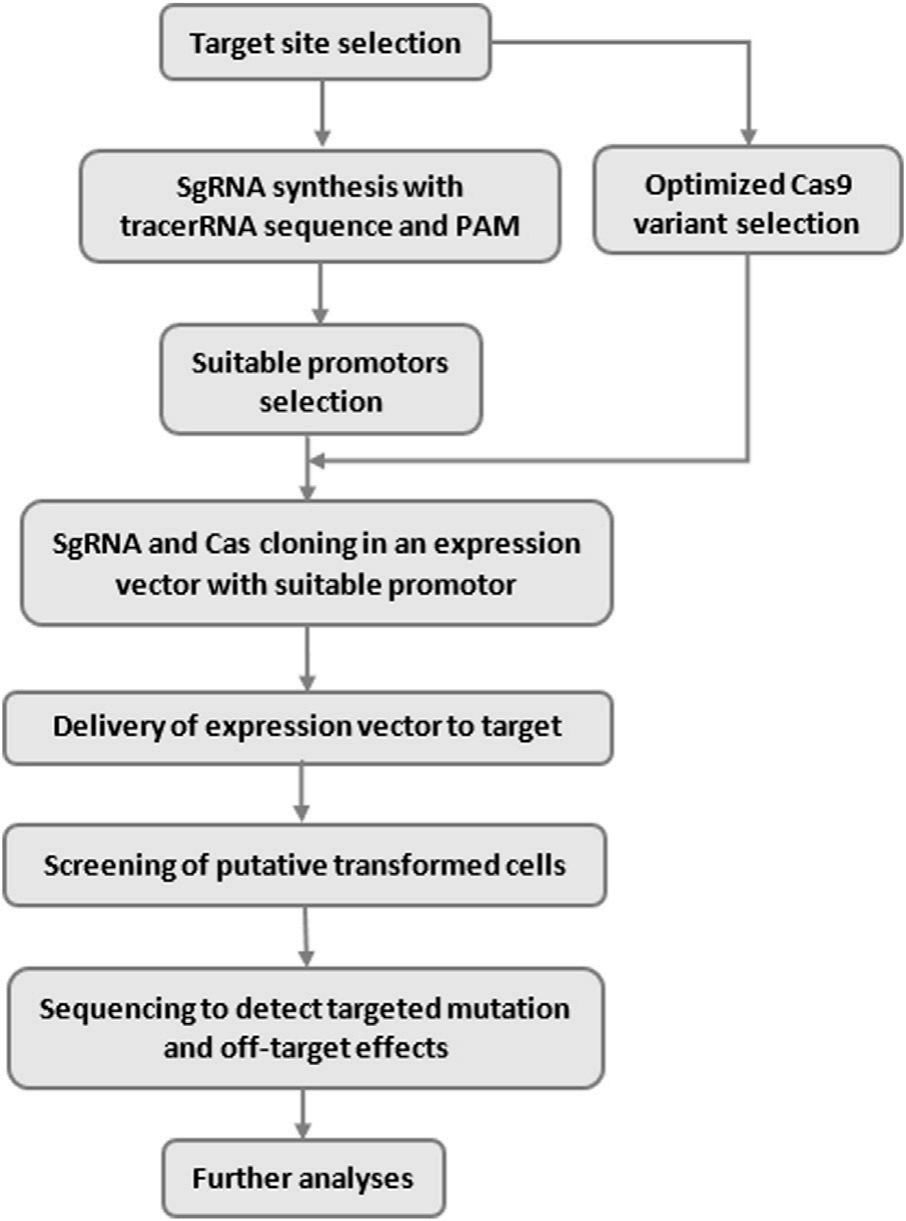
Basic overflow of CRISPR systems.

Over the last decade, the Clustered regularly interspaced short palindromic repeats (CRISPR)/Cas system has become the dominant tool for genome editing due to its simplicity, high performance, accuracy, and programmability (Gaj et al., 2013; Jacquin et al., 2019; Afzal et al., 2020). In addition, other influential factors such as ease of use, low cost, high speed, multiplex potential, and higher specific DNA targeting ability have increased the success and popularity of CRISPR across the global scientific community (Mali et al., 2013). The unique characteristics of this technology have made it one of the broad topics in molecular biology, synthetic biology, and genetic engineering (Jinek et al., 2012). Gene activation (CRISPRa), gene repression, CRISPR interference (CRISPRi), and epigenome editing are popular tasks in genome engineering using CRISPER. The basic overflow of the CRISPR systems is illustrated in Figure 1.

**FIGURE 2:**
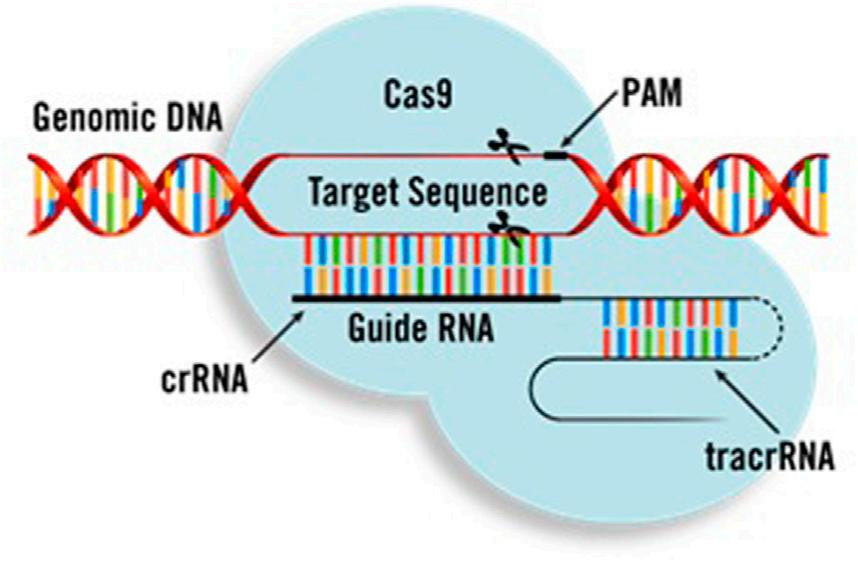
Main components of CRISPR (Duan et al., 2021).

As shown in Figure 2, CRISPR systems have three main components. The first one is a short synthetic guide RNA sequence (gRNA) necessary for Cas binding. The gRNA targets the Cas9 endonuclease (a protein which can cleave the DNA sequences) to define DNA. The gRNA can be supplied as a two-part system consisting of crRNA and tracrRNA, or as a single guide RNA (sgRNA), where the crRNA and tracrRNA are connected by a linker. The target’s recognition is facilitated by the protospacer-adjacent motif (PAM). Cleavage occurs on both strands 3 bp upstream of the PAM.

To use CRISPR for genome engineering, we need to select two components: Cas9 and gRNA (Gasiunas et al., 2012; Cox et al., 2015). Once a genome modification is decided, the first step is to identify the best site/sites for targeting Cas-induced DSBs (Jinek et al., 2014). The second step is to design the appropriative gRNA (Cui et al., 2018).

After designing gRNA, the only requirement for cleaving a CRISPR target site is finding a 3-base pair (3 bp) PAM. The form of PAM varies depending on the bacterial species of the Cas9 gene. For example, the most commonly used Cas9 nuclease, derived from S.pyogenes, recognizes a PAM sequence of NGG (Rabinowitz et al., 2020). Using the frequency of “GG” = 5.21% in the reference human genome, there would be an expected 161,284,793 NGG PAM sites in the human genome, or roughly one “GG” dinucleotide every 42 bases. So, cleaving unwanted sites, called off-target sites, is very common (Duan et al., 2021). Therefore, CRISPR target sites should be selected in such a way that minimizes potential off-target cleavage (Herai, 2019; Rabinowitz et al., 2020). But this is not always straightforward as it is not guaranteed that the desired cleaves will appear on just the selected site. Unfortunately, the existence of these unwanted cleaves is possible in every experiment. Therefore, activity (on-target) and specificity (off-target) are two critical factors considered when designing a genomic edition with CRISPR (Herai, 2019).

**FIGURE 3:**
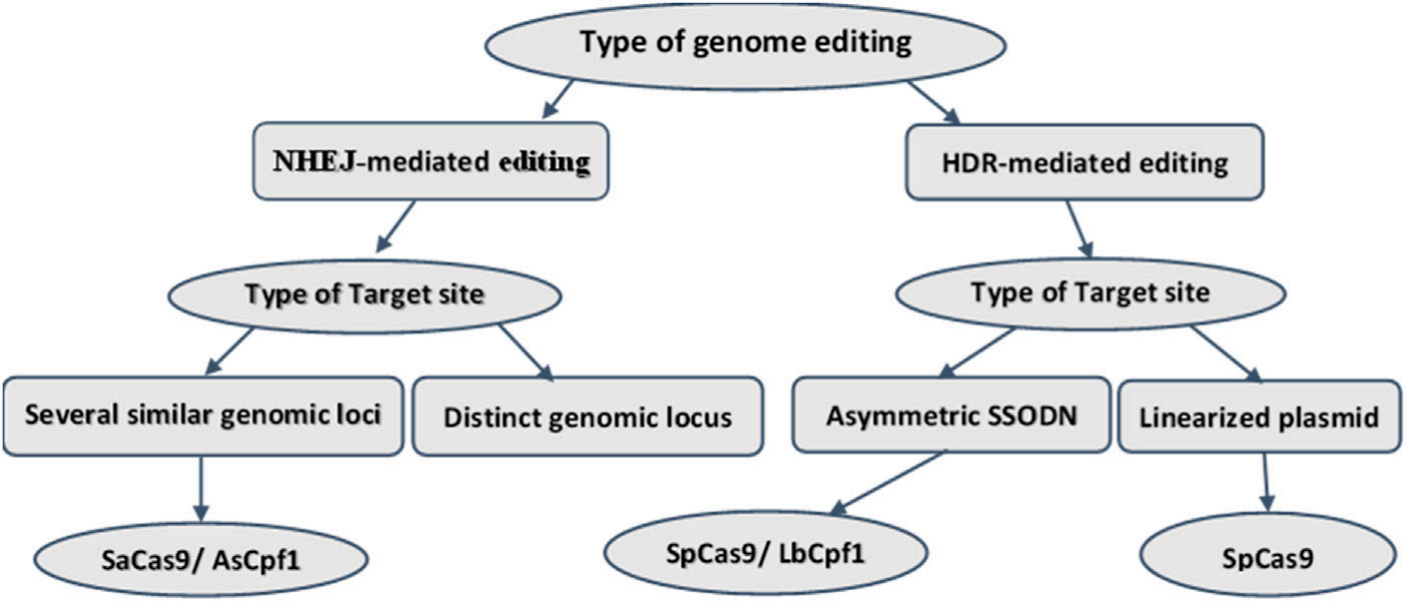
Selection of Cas enzyme.

According to research, the accuracy of CRISPR-based genomic edition depends on two issues: 1) the choice of Cas enzyme with suitable cutting power, 2) the choice of the appropriate cutting site, which relies on the performance of the gRNA. To achieve this, in the first step, we must select the optimal gRNAs contains high on-target activity and low (no) off-target efficiency (Moreno-Mateos et al., 2015; Luo et al., 2019; Manibalan et al., 2020). We will discuss this issue later. In the second step, we must select a suitable Cas enzyme [15]. In recent years, different variants of the Cas enzyme have been discovered. We can proceed according to Figure 3 to choose the proper Cas, depending on the type of editing. The choice of the Cas enzyme is effective on the PAM and the gRNA design.

In recent years, researchers have taken two main approaches for designing gRNAs, including experimental and machine learning-based methods (ML) (Lin and Luo, 2019). ML-based methods utilize the results of computational algorithms trained with real data to predict the effects of gRNAs instead of designing an actual experiment. Experimental methods are very costly and time-consuming (Chuai et al., 2017; Lin and Luo, 2019). In contrast, ML models are inexpensive and manageable. However, in terms of accuracy, they are still very different from experimental methods (Höijer et al., 2020). The accuracy of ML methods is highly dependent on the training process and the availability of adequate training data. Recent advances in the genome-wide analyses help researchers to discover all off-target sites, while the detection methods like Polymerase Chain Reaction (PCR) based methods, cannot find all of these sites. Using new sequencing technology, such as next-generation sequencing (NGS), and third generation sequencing which based on long-reads, can help us to detect more off-target sites. Mainly, single-molecule real-time sequencing (SMRT), has shown promising performance in genome sequencing. Researchers use these techniques to find more accurate information about off-target sites and use them in training their computational models (Lin and Wong, 2018; Höijer et al., 2020). Also, there are some repetitive, low complexity, AT/GC-rich regions, known as dark, in which ML-based tools cannot predict on-target and off-target sites in these areas. But amplification-free long-read sequencing technology helps to reveal Cas9 target sites even in these dark regions (Höijer et al., 2020). As the number of available features about on-target and off-target sites and the creation of large databases in this field increases, the predictions of ML-based methods become closer to experimental observations (Jiang et al., 2016; Abadi et al., 2017).

Some recent research has shown that ML-based methods can determine the extent of effective interactions and side-effects (changing unwanted sites) of each gRNA precisely (Abadi et al., 2017; Lin and Wong, 2018). Such a process can significantly accelerate the process of gRNA design for any part of human DNA, thus allowing us to edit anywhere in DNA (Jiang et al., 2016). However, existing models still have challenging issues, such as data imbalance, data heterogeneity, insufficient training data, generalizability, and cross-species inefficiency (Chuai et al., 2017).

We described the basic concepts of CRISPR systems and introduced activity and specificity as two main challenges in this area (Moreno-Mateos et al., 2015; Herai, 2019). In the rest of the paper, we provide an overview of computational approaches, especially machine and deep learning (MDL) algorithms, which we believe are the most effective and reliable methods for predicting gRNAs effects. The summary of our review is presented in Tables 1–Tables3, only for tools with active access link. Table 1 illustrates computational tools and software packages related to CRISPR systems; Table 2 summarizes tools and software packages related to finding off-target sites; Table 3 shows those related to gRNA design; and finally, Table 4 reports MDL-based tools and software packages related to CRISPR systems.

**TABLE 1:** Tools and software packages related to CRISPR systems.

Name	Main functionality	Input	Cell type	Interface	Year	Source
CRISPRidentify Mitrofanov et al. (2021)*	It detects All possible CRISPR arrays	Genome sequences	Bacteria and archaeal	Standalone application	2021	https://github.com/BackofenLab/CRISPRidentify
CRISPRloci Alkhnbashi et al. (2021)*	Definition of CRISPR leaders for each locus; Prediction of all CRISPR arrays in the correct orientation; annotation of Cas genes and associated information, include the Cas subtypes	Protein, genomic DNA, CRISPR repeats or viral sequences are accepted	Bacteria, archaeal and viral	Webserver and standalone versions (Python, Perl and Java)	2021	Webserver: https://rna.informatik.unifreiburg.deCRISPRloci
						Standalone version: https://github.com/BackofenLab/CRISPRloci
ANNOgesic Yu et al. (2018)	It can detect several genomic features, including genes, CDSs, tRNAs, rRNAs, TSSs, PSs, transcripts, terminators, UTRs, sRNAs, sORFs, circular RNAs, CRISPR-related RNAs, riboswitches, and RNA-thermometers	RNA-seg	Bacterial and archaeal genome	Command-line (Python)	2018	The software: https://pypi.org/project/ANNOge/ https://hub.docker.com/r/silasysh/annogesic/Documentation: http://annogesic.readthed.ocs.io/
CRISPR-DAV Wang et al. (2017)	A pipeline to analyze the CRISPR NGS data in a high-throughput manner. Output: read counts in various stages; read depths and indel frequencies in amplicon; counts and percentages of indel reads; frequencies of allele, SNP and HDR.	Files that describe software paths, parameters, mplicon, CRISPR sites, and FASTQ sources	Any selected genome	Command line Interface (Perl and R)	2017	https://github.com/pinetree1/crispr-dav.git and https://hub.docker.com/r/pinetree1/crispr-dav
Cas-analyzer Park et al. (2017)*	It is an NGS data analyzer. It categorizes and sorts the results. The position and size of insertions or deletions are depicted as interactive graphs	Deep sequencing data	Any selected genome	Web user interface (JavaScript)	2017	http://www.rgenome.net/cas-analyzer/
CRISPRAnalyzeR Winter et al. (2017)*	An application to analyze, document, and explore pooled CRISR/Cas9 screens. Reagent phenotypes such as efficiency scores and predicted genomic binding sites are displayed	An sgRNA library or screening data	Any selected genome	Open-source web or standalone application	2017	http://www.crispranalyzer.org
						source code at: http://www.github.com/boutroslab/CRISPRAnalyzeR
CRISPRcloud Jeong et al. (2017)	An application to extract, cluster, and analyze raw next-generation sequencing files derived from pooled screening experiments	sgRNA read counts data	Human and mouse	Cloud-based web application	2017	http://crispr.nrihub.org
CRISPRdigger Ge et al. (2016)	can Discover Direct Repeats (DRs) for CRISPRs and achieve a higher accuracy for a query genome	A genome sequence	Any selected genome	Command line application	2016	http://www.healthinformaticslab.org/supp/
BATCH-GE Boel et al. (2016)	It detects and reports indel mutations and other precise genome editing events and calculates the corresponding mutagenesis efficiencies	NGS-derived sequencing data, DNA of interest	Any selected genome	Command line application	2016	https://github.com/WouterSteyaert/BATCH-GE.git
CRISPRleader O’Brien and BaileyGT-Scan. (2014)	It detects leader sequences and shows full annotation of the CRISPR array and its strand orientation as well as conserved core leader boundaries	Genome sequence	Archaea and bacteria	Command line application (HTML pages)	2016	http://www.bioinf.unifreiburg.de/Software/CRISPRleader/
CRISPRDetect Biswas et al. (2016)*	It enables accurate identification of CRISPR arrays in genomes and their direction, repeat spacer boundaries, substitutions, insertions or deletions in repeats and spacers. It lists Cas genes that are annotated in the genome	Four inputs: genomic sequence, word size, min of word repeat, and max gap between repeats	Archaea and bacteria	Web application and command line (PERL)	2016	http://bioanalysis.otago.ac.nz/CRISPRDetect/
CRISPR-GA Güell et al. (2014)	It estimates the HR, NHEJ, and a complete report of the location and characteristics of the indels	The genomic region	Any selected genome	Web user interface (implemented in R)	2014	http://crispr-ga.net. Documentation at: http://crispr-ga.net/documentation.html
Crass Skennerton et al. (2013)	It identifies and reconstructs CRISPR loci from raw metagenomic data without the need for assembly or prior knowledge of CRISPR in the data set.	Raw file in FASTA or FASTq format	All genome	Command line interface	2013	http://bioinformatics.ninja/crass

**TABLE 2:** Tools and software packages related to finding off-target sites.

Name	Main functionality	Input	Cell type	Interface	Year	Source
CALITAS Fennell et al. (2021)	CALITAS is a CRISPR-Cas-aware aligner and integrated off-target search algorithm. It supports an unlimited number of mismatches and gaps and allows PAM mismatches or PAM-less searches	gRNA, one or more local regions of a target sequence	Human	Standalone application	2021	https://github.com/editasmedicine/calitas
CRISPR-SE Li et al. (2021)	It is an accurate and fast search engine using a brute force approach to find all off-target sites	gRNA	Human and mouse genomes	Web user interface	2021	The webserver: http://renlab.sdsc.edu/CRISPRSE/
						The source code: https://github.com/bil022/CRISPR-SE
CRISPRitz Cancellieri et al. (2020)*	It enumerates and annotates putative off-target sequences and assesses their potential impact on the functional genome. It has three outputs: i) all off-target sites; ii) an overall mismatch and bulge profile for each guide; iii) motif matrices	PAM sequence, a list of guides, References genome (required) and genomic annotations and number of mismatches (optional)	All genome	Standalone application	2019	https://github.com/pinellolab/CRISPRitz
						https://github.com/InfOmics/CRISPRitz
CHOPCHOP v3.0 Labun et al. (2019)	It Identifies sgRNA targets. Five outputs: i) the number of off-targets, ii) whether the off-targets contain mismatches or are perfect hits, and iii) where the target site lies within the gene iv) the results are ranked by GC-content	Four inputs: i) the target; ii) species; iii) CRISPR effector and iv) the purpose of the experiment	200 genomes	Command-line program and web user interface	2019	Server: https://chopchop.cbu.uib.no
						The local installation: https://bitbucket.org/valenlab/chopchop
CRSeek Dampier et al. (2018)	It finds all on-target and off-target sites	Interested sequence	All genome	Command line interface (Python)	2018	https://github.com/DamLabResources/crseek)
CRISPR-RT Zhu et al. (2018)*	It retrieves all the protentional targets and relevant information for gRNAs in CRISPR-C2c2 system	An RNA/DNA sequence	10 genomes include human	Web application	2017	http://bioinfolab.miamioh.edu/CRISPR-RT
PhytoCRISPEX Rastogi et al. (2016)	It finds potential targets and shows the gene name with start, stop, and sequence of the sgRNA targets. It also shows the results of checks at level one and two	DNA sequences	13 algae (diatoms, haptophytes, *etc.*), or any user defined genome	Web interface and UNIX-based standalone application	2016	http://www.phytocrispex.biologie.ens.fr/CRISPEx/crispexdownloads/
CRISPResso Pinello et al. (2015)*	It finds potential on and off-targets	Two files for paired-end reads or a single file for single-end reads, and the References amplicon sequence	Any selected genome	Command line interface or web server	2015	http://github.com/lucapinello/CRISPResso. Web application www.crispresso.rocks
Cas-OFFinder Bae et al. (2014)*	It searches for potential off-target sites and shows their locations, position, direction, and number of mismatches	Genome sequence	Any selected genome	Command line program (written in OpenCL) and website	2014	http://www.rgenome.net/cas-offinder
CasOT Xiao et al. (2014)	It finds potential off-target sites in any given genome with user-specified types of PAMs, and number of mismatches	target sites or genome and a genome annotation file (optional)	Any selected genome	Command-line program (a Perl script)	2014	http://eendb.zfgenetics.org/casot/
COSMID Cradick et al. (2014)*	It retrieves all off-target sites matching the user-supplied criteria in comparison to the guide strand with chromosomal location	The guide sequence, type of PAM, allowed number of mismatches, insertions and deletions, genome of interest, and primer design parameters	7 genomes including human and mouse	web user interface	2014	http://crispr.bme.gatech.edu
CRISPRdirect Naito et al. (2015)*	It outputs a list of on and off-target sites with details (target position, target sequence, the number of target sites in the genome, GC content, and calculated melting temperature)	Two inputs: i) an accession number, and ii) a genome coordinate or an arbitrary nucleotide sequence up to 10 kbp	9 genomes including human and mouse, rat *etc.*	Web user interface	2014	http://crispr.dbcls.jp
E-CRISP Heigwer et al. (2014)	It retrieves positions of CRISPR targets	Gene Id or gene sequence	More than 40 genomes	Web user interface	2014	http://www.e-crisp.org/E-CRISP
GT-Scan O’Brien and BaileyGT-Scan. (2014)	It ranks all potential on and off-targets	Genomic region and target rule (target length, constrained positions and positions with high-, low- or no-target and off-target specificity)	More than 25 genomes	Web user interface	2014	http://gt-scan.braembl.org.au
sgRNAcas9 Xie et al. (2014)*	It predicts all single or paired CRISPR target sequences and the corresponding information for each target site (such as start and end values, sequence pattern, GC content, sgRNA offset, *etc.*)	Sequences of target position	All genome	Command line interface (Perl script)	2014	www.biootools.com
SSFinder Upadhyay and Sharma. (2014)*	It identifies potential off-target sites and classifies them	File name and directory of input sequences	All genome	Command line interface (python)	2014	https://code.google.com/p/ssfinder/
CRISPRTarget Biswas et al. (2013)	It predicts the most likely targets of gRNAs. Targets can be displayed and scored for flanking sequences and PAMs	Spacers	Any selected genome	Web application	2013	http://bioanalysis.otago.ac.nz/CRISPRTarget

*Means the tools are free of charge to access.

**TABLE 3:** Tools and software packages related to gRNA design.

Name	Main functionality	Input	Cell type	Interface	Year	Source
SNP-CRISPR Chen et al. (2020)	It designs gRNAs for non-reference genomes to support allelic targeting. SNP-CRISPR calculates the gRNA efficiency score for the variant and the References sequences	Target genome, variant information including the genome coordinates and sequence changes	Human, Mouse, Zebrafish, Fly	Web application	2020	https://www.flyrnai.org/tools/snp_crispr/
AlleleAnalyzer Keough et al. (2019)	It designs allele-specific dual gRNAs. It incorporates single-nucleotide variants and short insertions and deletions to design sgRNAs for precisely editing one or multiple haplotypes of a sequenced genome, currently supporting 11 Cas proteins	Target genome (with genetic variant information)	Human	Application	2019	https://github.com/keoughkath/AlleleAnalyzer
CRISPR-Local Sun et al. (2019)	It designs sgRNAs in plants and other organisms that factor in genetic variation and is optimized to generate genome-wide sgRNAs	whole-genome sequencing, mRNA sequencing or known variants for specific transgenic receptor lines	Plants	Application	2018	http://crispr.hzau.edu.cn/CRISPR-Local/
CRISPR-P Liu et al. (2017)*	It helps to design of gRNA. It output: all targetable sites; the details and GC content of each gRNA; the restriction enzyme site in the targeting region; and synthetic DNA oligos; as well as the microhomology score and the secondary structure of sgRNA.	The gene locus tag, genomic position, or sequence	49 plant genomes	Web user interface	2017	http://cbi.hzau.edu.cn/crispr2/
CRISPR FOCUS Cao et al. (2017)*	It retrieves all possible gRNA and prioritize them. It also provides a rational and high-throughput approach for sgRNA library design	Gene symbols or RefSeq IDs	Human or mouse genome	Web application	2017	http://cistrome.org/crispr-focus/
Guide Picker Hough et al. (2017)*	It provides rapid guide RNA generation and selection. It retrieves guide sequences with on and off-target sites	The genome and the gene name	Mouse or human gene	Web application (JavaScript)	2017	https://www.deskgen.com/guide-picker/
SgTiler Ahmed and He. (2017)*	It generates graphical representation for distribution of sgRNA. It shows four outputs: i) all candidate sgRNAs; ii) list of filtered sgRNAs; iii) list of sgRNA details; and iv) a summary report with important statistics	Three input files: i) FASTA file; ii) A file with exon coordinates; and iii) a file of regulatory regions	Any selected genome	Command line application (Python)	2017	https://github.com/HansenHeLab/sgTiler
CRISPOR Concordet and Haeussler. (2018)	It finds guide RNAs in an input sequence and ranks them according to different scores. It evaluates potential off-targets in the genome of interest and predicts on-target activity	A sequence (typically an exon), a genome, and the type of CRISPR nuclease	More than 150 genomes	Web and standalone command line application	2016	http://crispor.org
CRISPR-DO Ma et al. (2016)	It retrieves information about target sequences, overlaps with exons, putative regulatory sequences and SNPs in the spCas9 CRISPR system	sgRNA	Human, mouse, zebrafish, fly and worm	Web application	2016	http://cistrome.org/crispr/
Breaking-Cas Oliveros et al. (2016)*	It retrieves all sequences, coordinates, scores, and annotation details of every gRNA and off-targets	The name of the References organism, the characteristics of the Cas-like nuclease, and the sequence(s) of the intended target genomic	All eukaryotic genomes	Web application	2016	http://bioinfogp.cnb.csic.es/tools/breakingcas
CT-Finder Zhu et al. (2016)	It helps users to design gRNAs optimized for specificity and shows Graphic visualization of on and off-target sites in Cas9n and RFNs	DNA sequence, a References genome, the on and off-target PAM sequences, and length of gRNA and seed region	Human, mouse, Arabidops	Web application	2016	http://bioinfolab.miamioh.edu/ct-finder
CRISPETa Pulido-Quetglas et al. (2017)	It helps to design sgRNAs	One or more target regions	Human, mouse, zebrafish, *Drosophila*, *melanogaster* and *Caenorhabditis elegans*	Command-line and web application	2016	Server: http://crispeta.crg.eu/manual Source code: https://github.com/guigolab/CRISPETA
CLD Heigwer et al. (2016)*	It helps to design sgRNAs	Three files: i) the genome sequence, ii) a parameter (Hwang and Bae, 2021) file, and iii) a gene list	All organisms	Command line application	2016	htts://github.com/
CRISPy-web Blin et al. (2016)*	It scans for gRNAs and potential off-targets	Target sequence or gene	Any microbial genome	Web application	2016	http://crispy.secondarymetabolites.org
EuPaGDT Peng and Tarleton. (2015)	It finds all gRNAs. It also scores, and ranks them. Additionally, it assists users in designing single-stranded oligonucleotides for homology-directed repair	Sequence or gene	Eukaryotic organisms	Web application	2015	http://grna.ctegd.uga.edu
Spacer Scoring for CRISPR(SSC) Xu et al. (2015)*	It predicts SgRNA efficiency	DNA sequence	Any selected genome	Web application	2015	http://crispr.dfci.harvard.edu/SSC/
Cas-Designer Park et al. (2015)*	It aids researchers in choosing appropriate target sites in a gene of interest. It outputs a list of all possible gRNAs and their potential off-target sites, including bulge-type sites, and also an out-of-frame score for each	DNA sequence	Most of genomes (Wang et al. (2019a)	Command line interface	2015	http://rgenome.net/cas-designer/
CRISPR multitargeter Prykhozhij et al. (2015)	It searches input sequences for single-sgRNA and two-sgRNA/Cas9 nickase targeting	sgRNA, GC%	12 genomes like zebrafish	Web application	2015	http://www.multicrispr.net/
CRISPR-ERA Liu et al. (2015)*	It designs gRNA. It outputs sgRNAs, on and off target location, and details of them with their E- and S-scores *etc.*	Target gene or genomic site	9 common prokaryotic and eukaryotic organisms	Web application	2015	http://crisprera.stanford.edu/InitAction.action
CCTop Stemmer et al. (2015)*	It identifies and ranks all candidate sgRNA target sites according to their off-target quality and displays full documentation	Target genome site	15 common prokaryotic and eukaryotic organisms	application (python)	2015	http://crispr.cos.uniheidelberg.de/
CRISPRseek Zhu et al. (2014)*	It identifies gRNAs and also scores and ranks them to minimize off-target cleavage	Any sequence	Any selected genome	Command line application ^®^	2014	http://www.bioconductor.org

*Means the tools are free of charge to access.

**TABLE 4:** MDL-based tools and software packages related to CRISPR systems.

Name	Main functionality	Input	Cell type	Interface	Model	Year	Source
C-RNNCrispr Zhang et al. (2020)	It predicts sgRNA on-target activity. It is a transfer learning approach by using small-sized datasets to fine-tune	Datasets to fine-tune	4 cell line	Standalone software	CNN and BGRU	2020	https://github.com/Peppags/C_RNNCrispr
CRISPRpred Muhammad Rafid et al. (2020)	It predicts sgRNA on-target activity	Position independent and position specific features	Human	Standalone software	SVM and random forest	2020	https://github.com/Rafid013/CRISPRpredSEQ
DeepCpf1 Kwon et al. (2019)	It predicts the activity of AsCpf1 (location of all targetable sequences and efficiency of each; information on GC contents, positions, strands, and DeepCpf1 scores.)	Cell line types, information on the sequences of a target and its surroundings, and References sequences	All genome	Web tool	CNN	2019	http://deepcrispr.info/
DeepHF Wang et al. (2019a)	It predicts SpCas9 activity for each gRNA (all targetable sequences, restriction sites, strands, and predicted efficiency)	Various types of SpCas9 nucleases, DNA sequences	All genomes	Web tool	CNN	2019	http://www.DeepHF.com/
CINDEL Iyombe. (2019)	It predicts the indel frequencies of CRISPR/Cas12 with TTTV PAM sequence (targetable sequences, positions, strands, GC contents, and INDEL scores)	References sequences		Web tool	-	2019	http://big.hanyang.ac.kr/cindel
DeepSpCas9 Kim et al. (2019)	It predicts SpCas9 activity for each gRNA (positions, GC content, and DeepSpCas9 scores)	Target sequence information with its surroundings, and gene symbols	Human	Web tool	CNN	2019	http://deepcrispr.info/DeepSpCas9
Microhomology-Predictor Hwang et al. (2021)	It predicts the deletion patterns by calculating the scores of possible deletion patterns produced by a MMEJ pathway following DNA cleavage by ZFNs, TALENs, or Cas9. All possible deletion patterns and the pattern scores can be checked	Target sites with high out-of-frame scores	All genome	Web tool	-	2019	http://www.rgenome.net/mich-calculator
inDelphi Cloney. (2019)	It predicts the spectrum of cut-site, possible sgRNA sequences, predicted mutation patterns, possible frameshift codons, and their frequencies	Sequences of both sides of cleavage in various cell types	Human and mouse	Standalone software	-	2019	https://indelphi.giffordlab.mit.edu
FORECasT Allen et al. (2019)	It predicts editing outcomes (possible mutation patterns and predicted frequencies of the mutation patterns and frame shifts) of the CRISPR/Cas9 system with NGG PAM.	Target DNA sequences and the cleavage sites	Most of genomes	Web tool	-	2018	https://partslab.sanger.ac.uk/FORECasT
CRISPR-GNL Wang et al. (2019b)	It is an algorithm for CRISPR on-target activity prediction	Normalized gene editing activity from 8,101 gRNAs and 2,488 features	human, mouse, zebrafish*Drosophila*, *Cioa intestinalis*	Stand alone application	regression models	2019	https://github.com/TerminatorJ/GNL_Scorer
DeepCRISPR Chuai et al. (2018)	It predicts whole genome on and off-target profiles	sgRNA sequences with an NGG PAM	Human	Web tool	CNN	2018	http://www.deepcrispr.net/
TUSCAN Wilson et al. (2018)	It predicts the degree of CRISPR/Cas9 activity and classifies them into active and inactive categories		All genome	Software	Random forest	2018	https://github.com/BauerLab/TUSCAN
SgRNAScorer Chari et al. (2017)	It identifies sgRNA sites and their activities for any PAM sequence of interest	Sequence with a defined spacer length and PAM sequence	Human and mouse	Web tool	SVM	2017	https://sgrnascorer.cancer.gov/
CRF Wang and Liang. (2017)*	CRF uses a classifier to filter out invalid CRISPR arrays from all putative candidates	DNA/RNA sequence in FASTA format	Bacteria and archaea	Web tool	Random forest	2017	http://bioinfolab.miamioh.edu/crf/home.php
GE-CRISPR Kaur et al. (2016)	It predicts and analyses sgRNAs efficiency and gives information like secondary structure of sgRNA, PAM, start and end of coordinates, and GC%	Desired gene or genome sequence in FASTA format	In any trained model		SVM	2016	http://bioinfo.imtech.res.in/manojk/gecrispr/
CRISPRscan Moreno-Mateos et al. (2015)	It’s a predictive sgRNA-scoring algorithm that captures the sequence features affecting the activity of CRISPR/Cas9 *in vivo*	DNA sequence	Fish	Web tool	Linier regression	2015	http://www.crisprscan.org/
WU-CRISPR Wong et al. (2015)	It predicts potential sgRNAs and scores of them	Gene IDs	Human and mouse	Web tool and stand-alone software	SVM	2015	http://crispr.wustl.edu
SSC Xu et al. (2015)	It’s a program for predicting editing activity of SpCas9 and giving all possible targets with the efficiency scores of various editing modes such as knockout, CRISPRi, or CRISPRa	Target sequences with the length of spacers (19 nt or 20 nt) as		Web tool	Elastic Net	2015	http://cistrome.org/SSC/
CRISPRstrand Alkhnbashi et al. (2014)	It determines the crRNA-encoding strand at CRISPR loci by predicting the correct orientation of repeats. It also determines whether repeats lie on the forward or reverse strand	Attribute type, attribute order, size of the terminal regions, number of blocks within the terminal regions	Bacteria and archaea	Integrated in CRISPRmap web server	graph kernels	2014	http://rna.informatik.uni-freiburg.de/CRISPRmap

*Means the tools are free of charge to access.

## 2 Computational approaches in CRISPR

Computational approaches are an essential part of CRISPR research. The bioinformatics studies have made significant contributions to the initial discovery of CRISPR (Alkhnbashi et al., 2014; Makarova et al., 2015). We summarize some of them in Table 1. Bioinformatics tools play a significant role in these fields: 1) determination of the specific differences between the CRISPR/Cas systems from archaeal and bacterial sources; 2) determination of required repeat spacer sequences for processing the mature CRISPR RNA (crRNA); 3) prediction of the transcribed strand of CRISPR arrays; 4) determination of CRISPR leader sequences; 5) classification of Cas proteins; 6) prediction of proper gRNA; 7) prediction of on-target and off-target effects; and so on (Listgarten et al., 2016; Lin and Wong, 2018; Listgarten et al., 2018; Herai, 2019; Alkhnbashi et al., 2020; Smith et al., 2020).

According to our review, low cleavage efficiency and off-target effects hamper CRISPR development and application. So, prediction of proper gRNA and prediction of on-target and off-target effects is so critical. In the rest of the paper, we will focus on the tools that have been developed for designing optimal gRNA with low off-target effects.

### 2.1 gRNA design

There are two fundamental questions in CRISPR researches. The first question is: what are the targets of the given gRNA? Some methods, such as CRISPResso (Pinello et al., 2016) and CRISPRTarget (Biswas et al., 2013), try to calculate potential targets by taking a gRNA as input and using computational algorithms (more details are described in Table 3). Tools like CRISPRTarget (Biswas et al., 2013) offer a way to answer this question using a ML-based approach (Table 4 shows more details). The second important question is how to be confident about the accuracy of CRISPR edits. Most of the tools or methods in CRISPR’s field have been developed to answer these two questions. In Tables 2, 3, we tried to collect all of them and describe their details.

Also, we realized that most of researches in CRISPR area mainly focus on increasing cleavage activity (more on-targets) and cleavage efficiency (low off-target sites). As known, low efficiency makes CRISPR editing unreliable and also hampers CRISPR development and application (Wang et al., 2019a). Unfortunately, the high focus on more activity induces more off-target cleavage, which can be toxic. Therefore, we must maintain a balance between these two criteria. These issues can be resolved by designing successful CRISPR gRNA and choosing an appropriate Cas protein (Kuscu et al., 2014; Shen et al., 2018).

As mentioned earlier, cleavage efficiency varies significantly among different target sites and cell lines (Yan et al., 2018). Several features can influence the gRNA binding ability and the Cas enzyme cutting efficacy. Sequence composite features (nucleotide position, GC content), genetic and epigenetic features (chromatin accessibility, gene expression), and energetic properties (RNA secondary structure, melting temperature, free energy) are the most important influential features on cleavage efficiency (Pallarès Masmitjà et al., 2019; Wang et al., 2020). Based on these features, many computational tools have been developed for designing highly efficient gRNAs. In the rest of this section, we will discuss the most popular ones.

Rule set 1 (Liu et al., 2020) is a ML-based model that uses a support vector machine (SVM), a supervised ML method, and contains a linear regression method for classifying gRNAs. Rule set 1 uses sequence-based features, and its predictive data is highly correlated with experimental results (Xu et al., 2015). Rule set 2 (Liu et al., 2020) is an improved version of Rule set 1 and counts the nucleotides independent location of the gRNA target site within the gene to improve results (Doench et al., 2016). It is a powerful model, used for both CRISPR Knock Out (CRISPR KO) and CRISPR activation/interference (CRISPRa/i) experiments. Another powerful model-based package has been developed and implemented at the Broad Institute to predict gRNA efficiency, named sgRNA Designer (Pallarès Masmitjà et al., 2019).

Elastic Net is another ML-based and regularized regression-based method (Li and Lin, 2010). Although there are significant differences in nucleotide preference between CRISPR KO and CRISPRa/I, the Elastic Net algorithm is used to construct models for both CRISPR KO and CRISPRa/i. Also, this practical algorithm has been applied in Spacer Scoring for CRISPR (SSC) software to predict the gRNA efficiency (Qin et al., 2019). Additionally, well-known platforms such as E-CRISP (Heigwer et al., 2014), CHOPCHOP (Labun et al., 2019), and CRISPRFOCUS (Cao et al., 2017) have applied this method.

Moreno and his colleagues designed another logistic regression-based method and integrated it into CRISPRscan to predict the gRNA precision (Moreno-Mateos et al., 2015). Additionally, they have applied extra features such as guanine enrichment and adenine depletion, which increase the gRNA activity (Cui et al., 2018).

Another ML-based method is WU-CRISPR (Wong et al., 2015) which uses sequence composite features like guanine enrichment and adenine depletion, and some other novel features to build a higher precision model. The CRISPR/Cas9 target online predictor (CCTop) (Stemmer et al., 2015), a platform for CRISPR target prediction, takes advantage of this model. The SgRNAScorer is another software that uses SVM to calculate gRNA on-target scores. The new version of this software can predict other Cas systems such as SaCas9 (Qin et al., 2019) and AsCpf1 [94].

To avoid unwanted effects in other sites except for desired target sites (off-target), researchers try to modify a spacer sequence that does not adopt other sites in the genome. Tools such as CRISPRpred (Hwang and Bae, 2021), DeepSpCas9, and SgRNAScorer are usually limited to the set of preprocessed genomes used when training ML models. To build good gRNAs in genomes other than those used in the training process, researchers can use web-based tools such as CRISPy (Blin et al., 2016). Looking at Tables 1–Tables 4, we have listed the genome in which the editing takes place (named target genome) as a significant feature for all tools. The existence of target genome is even more critical for deep learning-based (DL) methods, because they are usually unpractical in genomes other than the ones from which training data was extracted. Basically, being used in all genomes is a significant strength for ML-based tools. But one tool may not have the same accuracy over all genomes or even all regions of a genome (see Figure 7) (Kim et al., 2021). Furthermore, structural correctness and base-level accuracy of the target genome are important. The accuracy of a genome differs not only between genome sequencing technologies but also across genomic regions, as some stretches of the genome are inherently more difficult to read (Kim et al., 2021). It is commonly known that certain genomic regions are more difficult for sequencing and extracting features. AT-rich or GC-rich regions, which are important for detecting off-target sites, are tough because they respond poorly to the amplification protocols required by some platforms. Palindromic sequences or hairpin structures similar to gRNA structures are difficult to denature, making such regions challenging for sequencing tools (Selvakumar et al., 2022).

#### 2.1.1 Selecting the best gRNA

There may be several gRNAs for an experiment, in which case we have to pick the best one. Many computational approaches have been developed for scoring and selecting the best gRNAs. Some of them use experimental data to score a gRNA. According to the different criteria, these methods consider a specific score for each gRNA. The criteria and final score calculation are different in each algorithm. CHOPCHOP (Labun et al., 2019) provides multiple scores for users, such as Rule Set 1 and Rule set 2, SSC (Xu et al., 2015), CRISPRscan [13], and deepCpf1 (Kim et al., 2018). E-CRISP (Heigwer et al., 2014) uses a particular score to determine the quality of each gRNA, named SAE, which combines three scores: specificity, annotation and efficacy. E-CRISP uses Rule Set 1 and SSC too. CCTop (Stemmer et al., 2015) calculates the CRISPRater score to predict the efficiency of gRNAs. CCTop also calculates off-target scores for each sequence. The CRISPOR (Concordet and Haeussler, 2018) ranks gRNAs according to different scores, such as on-target activity and protentional off-targets scores.

To score a gRNA or determine whether it is suitable for the desired genome editing or not, we need to determine potential targets of a gRNA in the selected genome and determine which of these potential targets are desirable. Hence, the number of on-target and off-target sites is critical in gRNA evaluation. In other words, since genomic edits are permanent and very sensitive, it is crucial to determine potential targets before the main editing occurs and then remove or reduce them (Yan et al., 2018). Therefore, many researchers have focused on this issue. Furthermore, many developers have attempted to develop practical tools for this purpose. We will discuss these tools in the next section.

### 2.2 Prediction of CRISPR specificity (off-target sites)

The prediction of off-target mutations in CRISPR/Cas9 is a hot topic owing to its relevance to gene-editing research. Cas nucleases may cleave unintended genomic sites and cause unexpected mutations called off-target cleavage (Listgarten et al., 2018). Even though the CRISPR/Cas9 system is routinely used in a large variety of tasks, there is also a significant concern that off-target effects may reduce its effectiveness of CRISPR. In response to this concern, researchers have concluded that the best way to mitigate off-target effects is to know when and where they occur and then design guides to avoid them while balancing for on-target efficiency. By predicting CRISPR cutting specificity and designing optimal gRNAs, off-target effects can be effectively relieved. As noted earlier, careful CRISPR target selection and low concentrations of CRISPR components can reduce off-target cleavage (Zetsche et al., 2020).

The off-target predictive modelling problem can be broken down into three main tasks. Given a gRNA to evaluate off-target activity, one needs to (Afzal et al., 2020) search the whole genome for potential targets; in other words, search those regions of the genome matching the guide sequence with up to X number of mismatches (Gaj et al., 2013); score each potential target found in step 1 according to its activity (Jacquin et al., 2019); collect the second stage scores and evaluate the final score of a gRNA. Several solutions have been presented for these tasks, including Cas-OFFinder (Bae et al., 2014), CRISPOR (Concordet and Haeussler, 2018), CHOPCHOP (Labun et al., 2019), and e-CRISPR (Tarasava et al., 2018). These models differ in their search algorithms and the completeness of the search process. Completeness is dictated by options such as the maximum number of mismatches, allowed PAMs, and the search algorithm used.

**FIGURE 4:**
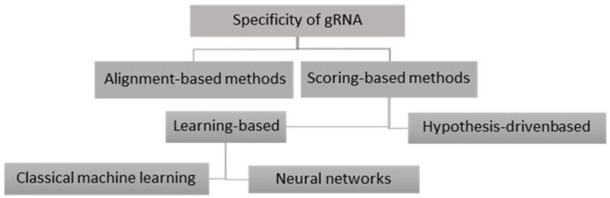
Basic methods to predict the specificity of CRISPR gRNAs.

There are two basic methods to predict the specificity of CRISPR gRNAs: the alignment-based and the scoring-based methods. In the following, we will explain these approaches and give successful examples of each one. Also, the overview of these approaches is depicted in Figure 4.

#### 2.2.1 Alignment-based methods

In the alignment-based method, gRNAs are aligned to a given genome, and off-target sequences and sites are returned. These methods are mainly used to find out all potential off-target sites *in silico*. Choosing a search engine and setting search parameters plays an important role in evaluating these tools (Liu et al., 2020). For example, if we set the maximum number of mismatches to a large number, like four or more, we will probably find all possible off-targets. The observed rate of off-target activity is about 59% when there is one mismatch between the target DNA and gRNA sequences and decreases toward 0% when four or more mismatches exist (Kim et al., 2021). So, it can be concluded that an increased number of mismatches decreases the likelihood of off-target activity.

Common sequence alignment tools use BLAST, BLAT, Bowtie, Bowtie2, BWA or customized search engines. Table 5 summarizes the search engine of famous alignment-based tools in CRISPR.

**TABLE 5:** The most popular alignment-based methods and related search engines.

Search engine	Methods
BLAST	CRISPRTarget (Biswas et al., 2013), CRISPR-P (Liu et al., 2017), and CRISPR-GA (Luyten et al., 2004)
BOWTIE	CRISPR-ERA (Liu et al., 2015), CHOPCHOP (Labun et al., 2019), CasFinder (Upadhyay and Sharma, 2014), CCTop (Stemmer et al., 2015), E-CRISP (Heigwer et al., 2014), and CLD (Heigwer et al., 2016)
BWA	CRISPR-DO (Ma et al., 2016), CRISPOR (Concordet and Haeussler, 2018), and CRISPETa (Pulido-Quetglas et al., 2017)
BRUTE FORCE	GuideScan (Perez et al., 2017), Cas-OFFinder (Bae et al., 2014), FlashFry (McKenna and Shendure, 2018), Crisflash (Jacquin et al., 2019), CRISPRitz (Cancellieri et al., 2020), and CRISPR-SE (Li et al., 2021)

Compared to methods which use BLAST, Bowtie and BWA as search engine, methods like GuideScan (Perez et al., 2017), Cas-OFFinder (Bae et al., 2014), FlashFry (McKenna and Shendure, 2018), Crisflash (Jacquin et al., 2019), CRISPRitz (Cancellieri et al., 2020), and finally, CRISPR-SE (Li et al., 2021)are faster due to the use of Brute force search engine. In addition, unlike most methods that support only a limited number of mismatches (mostly 3 or 4), Cas-OFFinder, CRISPRitz and CRISPR-SE have more preference due to their support of any number of mismatches.

The Bowtie and BWA are traditional tools for short sequence alignment that can be used for off-target sites detection (de Ruijter and Guldenmund, 2016). However, they cannot identify small PAMs since they were developed for NGS read alignment. Moreover, these tools allow very limited mismatches with default parameters, so they cannot identify all potential off-target sites.

Most tools, like CCTop (Stemmer et al., 2015), modify default algorithms and parameters and utilize Bowtie (de Ruijter and Guldenmund, 2016) to find off-target sites. CCTop follows three main steps. In the first step, CCTop identifies PAM sites; In the second step, it modifies default parameters (up to five mismatches against one in default) of Bowtie, and uses them to search for matches and mismatches in protospacer sequences. In the third step, it evaluates the off-target score for each candidate gRNA.

SeqMAp (Jiang and Wong, 2008) is an ultrafast short sequence mapping tool used in sgRNAcas9 (Xie et al., 2014) to find off-target sites. The sgRNAcas9 classifies all off-target sites into three categories and scores them to choose the best gRNA.

CasOT (Xiao et al., 2014) is another tool that can find Cas9 on-target and off-target sites with up to six mismatches in the seed region (12 nucleotides adjacent to the PAM). This tool can also determine whether off-targets are within a coding exon (Listgarten et al., 2016) or not. FlashFry (McKenna and Shendure, 2018) is another alignment-based method that defines off-targets with high speed. Additionally, it chooses the best gRNA and provides useful information such as annotating off-target sites, on and off-target scores, GC content, *etc.* FlashFry is a good choice for many applications because of its high speed and comprehensive output. Crisflash (Jacquin et al., 2019) is another one that belongs to the alignment-based approaches group. Crisflash designs gRNAs with a tree-based algorithm and uses user-supplied variant data to optimizes gRNA accuracy. It uses an N-ary tree structure, which searches up to four mismatches. CRISPRitz (Cancellieri et al., 2020) used a four-bit-based encoding to represent each nucleotide to allow for efficient bitwise operations. CRISPRitz supports off-targets with both mismatches and indels.

CALITAS (Fennell et al., 2021) is a new CRISPR-Cas-aware aligner tool which uses a modified and CRISPR-tuned version of the Needleman–Wunsch algorithm, supports an unlimited number of mismatches and gaps, and allows PAM mismatches or PAM-less searches. CALITAS returns a single best alignment for a given off-target site and it enables off-targets to be referenced directly using alignment coordinate.

CHOPCHOP v3.0 (Labun et al., 2019), a well-known model, is another tool that uses Bowtie with parameters–V and–L to detect off-target sites [90]. But, CRISPOR uses BWA to find all potential off-target sites iteratively and can find all validated off-targets as well as Cas-OFFinder (Bae et al., 2014).

Sequence alignment tools like CRISPy (Qin et al., 2019) and CRISPRdirect (Heigwer et al., 2016) rely on a minimum of one K-mer exact match. They are likely to miss some off-targets, spatially with a high number of mismatches and ultra-short gRNAs (20-mer). So, the accuracy of these methods cannot be very high.

In recent years, some tools like GuideScan (Perez et al., 2017), Cas-OfFinder (Bae et al., 2014), and CRISPR-SE (Li et al., 2021) have been developed with Brute force algorithm as their search engine. GuideScan uses a “tree” data structure with a brute-force algorithm that guarantees the search accuracy. Another tool in this category is Cas-OFFinder. Cas-OFFinder is one of the most popular tools for detecting potential off-target sites, with no limit to the number of mismatches, PAM types, or gRNA length. In our opinion, the most significant advantage of Cas-OFFinder is its high running speed due to using GPUs. It can also predict off-target sites with one-bp deletions or insertions.

OffScan (Cui et al., 2020) is the last one we considered in this study that is, belongs to the alignment-based approaches group. OffScan is not limited by the number of mismatches and allows custom PAM. Besides, OffScan adopts the FM-index, which efficiently improves query speed and reduce memory consumption.

Here, we discussed several alignment-based methods for the prediction of the gRNA output and realized that Cas-OFFinder may be the best option for identifying all potential off-targets with any Cas nucleases among these tools. Although users can reduce the number of outputs by restricting the maximum mismatches while exploring off-target cleavage, there are always redundant outputs; many are false positives.

On the whole, all nucleotide positions containing mismatches do not have the same decisive effect on off-target cleavage, but this issue is not considered in alignment-based methods. Because of this problem, and in order to increase the accuracy of the off-target detection methods, adding the features that influence the non-specific binding of CRISPR gRNAs to the methods is essential. As a result, another group of approaches emerged called scoring-based methods, which are discussed in the following sub-section.

#### 2.2.2 Scoring-based methods

In the scoring-based method, the gRNAs identified in the alignment process are scored and ranked, and the sgRNA with the highest score is selected. There are two groups of scoring-based approaches: 1) hypothesis-driven-based approaches, where off-targets are scored based on the contribution of specific genome context factors to gRNA specificity; 2) learning-based approaches, where gRNAs are scored and predicted from a training model that considers the different features affecting specificity.

MIT (Hsu et al., 2013) is the first popular score-based tool for CRISPR off-target evaluation. To score the off-target efficiency of each gRNA, it counts and evaluates the contributions made by different mismatch positions. It also calculates a weight matrix to determine off-target efficiency for each gRNA (Chuai et al., 2017). The MIT score has been integrated into many CRISPR gRNA design tools, such as CHOPCHOP v3.0 CHOP (Labun et al., 2019) and CRISPOR (Concordet and Haeussler, 2018).

Another popular score-based tool for off-target evaluation is CFD (Cutting Frequency Determination). It is noticeable that gRNA can bind genome loci with non-canonical PAMs such as NAG, NCG, and NGA. So, CFD has added PAM features to their scoring metrics (Abadi et al., 2017). Also, for examining correlations between RNAs and off-targets, gRNAs with mismatches and indels in target sequences are added. GUIDE-seq (Tsai et al., 2015) validated the CFD score and proved that it performs better than the MIT score. The CFD score has been integrated into CRISPRscan (Moreno-Mateos et al., 2015), GuideScan (Perez et al., 2017), CRISPOR (Concordet and Haeussler, 2018), and others. CRISPRoff (Carlson-Stevermer et al., 2020) and uCRISPR (Carlson-Stevermer et al., 2020) integrated energetic properties into their scoring metrics. They both yielded better accuracy than MIT and CFD in off-target prediction.

Scoring-based methods consider only a few features, and unfortunately, all practical features cannot be considered. Also, most features are not understood yet, while learning-based methods use combinations of multiple features to build complex models for better prediction of off-target sites. These models are based on ML and, more recently, DL methods.

DL-based methods are attractive for CRISPR gRNA target efficacy prediction. They are mainly based on CNNs. Table 4 introduces some famous models that use MDL models for gRNA on-target prediction. These models used neural networks to extract features from the input genomic sequence. Generally, they are superior to models that use classical ML tools in prediction accuracy.

DeepCRISPR (Chuai et al., 2018) is a DL-based platform that combines gRNA on-target and off-target site predictions. As mentioned, in DL-based models, we do not need to identify all effective features, as they are detected automatically using the deep neural network. DeepCRISPR learns all possible sequence and epigenetic features that may affect gRNA Knock Out (KO) efficacy (Hana et al., 2021) in its learning process with a large dataset that is, gathered for it.

CRISPR-Cpf1 (Kim et al., 2017) is a ML-based model that achieved high efficiency, although it suffers minor off-target effects. DeepCpf1 (Kwon et al., 2019) is another highly used DL-based algorithm, mainly used in predicting Cpf1 activity. It uses chromatin accessibility data. It showed a significant improvement in the accuracy of Cpf1 activity prediction. CRISPR-DT (Zhu and LiangCRISPR-, 2019) is a recently developed platform for predicting the Cpf1 target efficiency. This model has been implemented with the SVM algorithm and displays better performance than the DL-based models such as DeepCpf1.

CRISPOR (Concordet and Haeussler, 2018) may be the best tool for designing gRNAs. CRISPOR combines multiple tools and gathers a large dataset to develop a highly efficient CRISPR gRNA design. CRISPOR contains 417 genomes and 19 PAM types, making it useful in almost all genomes. CRISPOR calculates two specificity scores: MIT and CFD. Additionally, it calculates ten efficiency scores, including Rule Set 2, CRISPRscan, microhomology, Lindel scores (Chen et al., 2019) and others for outcome prediction. CRISPOR designs primers for each gRNA as well as off-target sites. These primers are helpful when conducting on and off-target validation. CRISPOR enables the filtering of gRNAs with genomic variants based on well-known variant databases.

Some computational tools use CNNs for feature extraction or classification of CRISPR Cas. For instance, Seq-deepCpf1 (Kim et al., 2018; Kwon et al., 2019) has used CNN to extract features from the input gRNA sequence. And DeepCRISPR incorporates a CNN for predicting CRISPR/Cas9 gRNA on-target knockout efficiency and whole-genome off-target profiles. Also, DeepCas9 uses CNN to automatically learn the sequence determinants and predict the activities of gRNAs across multiple species genomes (Bhagwat and Khuri, 2021). Deeper-Bind (Hassanzadeh and Wang, 2016) used a LSTM layer to learn the dependencies between sequence features; this helps improve the prediction of protein binding specificity (Zhang et al., 2020). C-RNNCrispr (Zhang et al., 2020) has used a hybrid architecture combining CNN with bidirectional GRU (BGRU) to predict sgRNA cleavage efficacy (Sledzinski et al., 2020).

**FIGURE 5:**
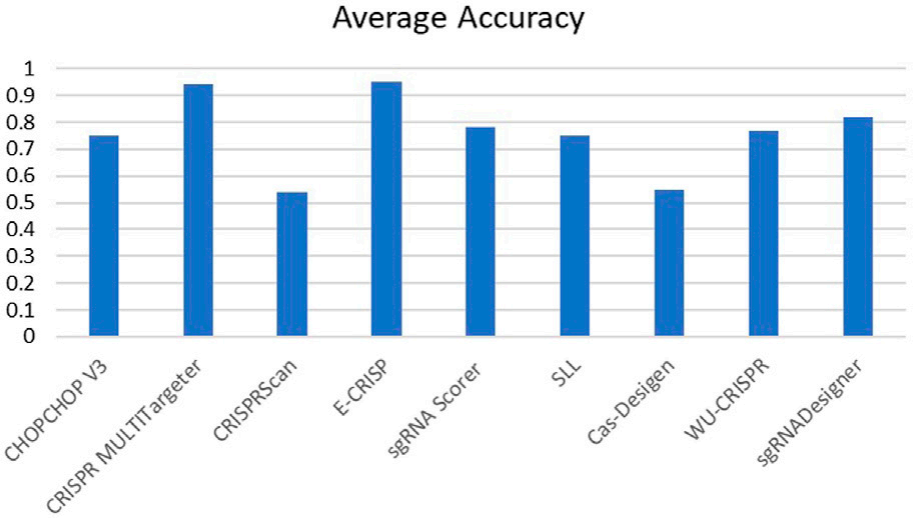
Average accuracy of off-target prediction.

**FIGURE 6:**
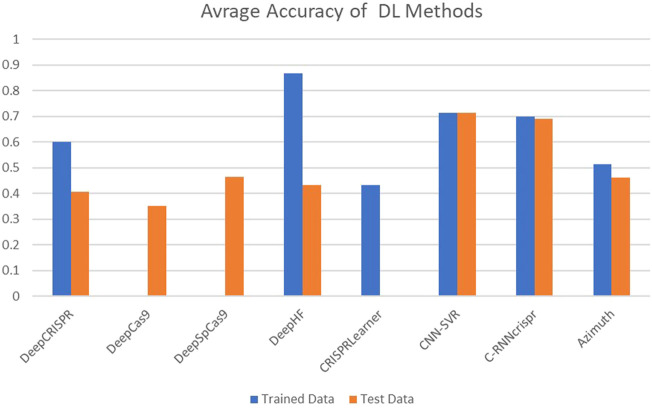
Average accuracy of off-target prediction in DL-based methods.

The performance of these tools is quantitatively assessed with two commonly used evaluation metrics, including accuracy and Spearman Correlation Coefficient (SCC) between predicted and real detected off-target activity. However, other evaluation metrics like Precision and Sensitivity (Eqs 2, 3) are used in some research as well. Spearman correlation seems to be a more reliable criterion. Most of these tools achieve promising accuracy in off-target prediction. Figures 5, 6 compare the off-target prediction efficacy of some popular tools. Due to their importance, we compare the accuracy of DL-based tools in separate diagram. The average accuracy of these tools is illustrated in the figures, as their accuracy differs among different genomes. For example, DeepCRISPR was the most accurate tool in the HEL cell line but performed poorly in the others. More details can be found in (Wang et al., 2019a; Zhu and LiangCRISPR-, 2019). Also, as a ML method, the accuracy differs between the train and test datasets. Unfortunately, for DeepCas9 and DeepSpCas9 (Chen et al., 2019), there is no report in their primary reference for the training dataset and the test dataset in CRISPRLearner (Bhagwat and Khuri, 2021). Accuracy, Precision, and Sensitivity are defined as follows, where TP, FP, TN, and FN represent true positive, false positive, true negative, and false negative, respectively.

$$Accuracy=\frac{TP+TN}{TP+FP+TN+FN}$$ (1)

$$Precision=\frac{TP\;}{TP+FP}$$ (2)

$$Sensitivity=\frac{TP\;}{TP+FN}$$ (3)

**FIGURE 7:**
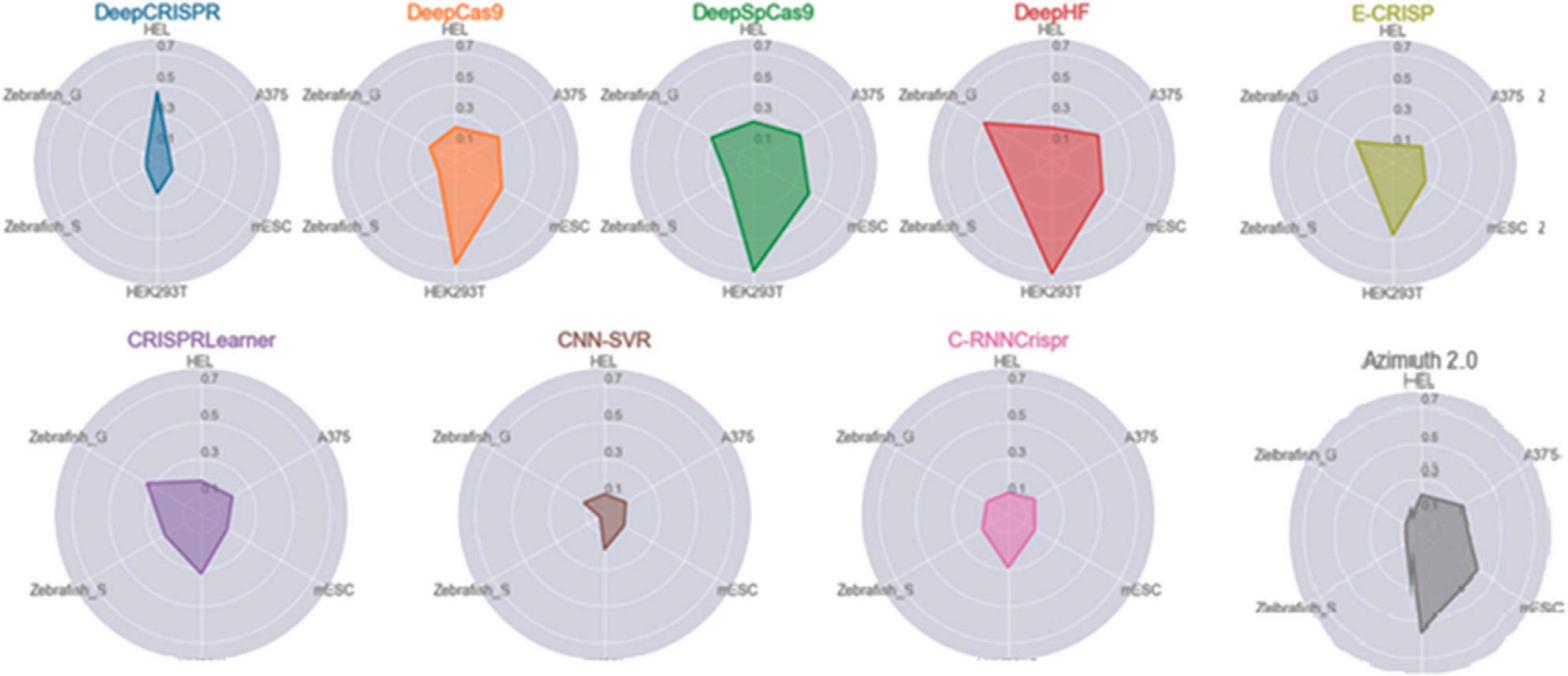
Spearman correlation for ML-based tools over the different datasets. Each polygon represents a tool, and the edges illustrate the obtained correlation over the respective dataset.

SCC evaluates the ability of the models to predict the actual efficiency of each gRNA sequence (Konstantakos et al., 2022). While some models are trained to minimize the mean squared error (MSE), the comparison between models on different datasets is necessarily made using Spearman correlation. Figure 7 compares the predictive ability of off-target sites in some ML-based tools over five datasets named Zebrafish_G, Zebrafish_S, HEL, A375, and mESC. In general, the larger the polygon area, the better the overall performance of the tool. Figure 7 clearly illustrates the better and more robust performance of the DeepHF, DeepSpCas9, and DeepCas9 models. As shown, classic ML-based tools such as Azimuth 2.0 achieve comparable performance to DL-based tools. Also, even though E-CRISP is more accurate than some learning-based tools, it does not achieve high enough correlations. However, E-CRISP has stable performance across all datasets. In addition, as it is clear from Figure 7, DeepCRISPR outperforms the other tools on the HEL dataset, and E-CRISP and CRISPRLearner achieve better results based on this metric.

As mentioned, gRNAs are typically designed by computational tools which compare gRNA sequence with a reference genome to predict the activity of on-target and potential off-targets. However, these tools can yield false-positive (FP) or false-negative (FN) results. Furthermore, the DNA in clinical experiments can differ from the reference genome used in the computational modeling, which means they would be more false predictions. Therefore, the accuracy is less than the values shown in Figure 7 in the actual experiment. To resolve this problem, *in-vitro* based tools have been developed for the experimental detection of off-target sites in a particular DNA sample. Tools like SMRT-OTS and Nano-OTS (Höijer et al., 2020) use long-read single-molecule sequencing.

In this article, we review both traditional and ML-based approaches for gRNA designing and predicting off-target sites. As mentioned before, experimental methods which use third-generation sequencing technology, have a better performance in Cas9 target detection on dark genomic regions (Höijer et al., 2020). This new technology helps us to detect more on-target and off-target sites and to design optimal gRNA. Furthermore, collected data in experimental methods, could improve the accuracy of DL-based tools.

Also, we have presented a comprehensive list of available tools. Each tool has merits and demerits, and the performance of different tools differs in different situations. According to our studies, some tools can be a better choice in some situations; However, others may be more popular in the scientific community. So, choosing the right tool depends on the conditions and limitations of an application.

Among the alignment-based methods, tools like CRISPR-P, Flycrispr, CRISPRseek, Cas-OFFinder, CasOT, sgRNACas9, and Flashfly have high accuracy and efficiency; however, CRISPR-P and Flycrispr are only used in specific genomes. Other tools such as CRISPRseek, Cas-Offinder, and CasOT, are used in almost all genomes. Moreover, they support only particular types of PAMs, while methods such as sgRNACas9 and Flashfly are compatible with all types of PAMs and seem to be a better option for designing gRNAs.

Among the learning-based methods, DL-based methods, including C-RNNCrispr, DeepCpf1, DeepHF, DeepSpCas9, and DeepCRISP, have drawn much interest recently. However, learning-based methods such as CLD, CRISPR-ERA, sgRNA-design, E-CRISP are significant due to their high accuracy and use in all genomes. Finally, based on our study, methods such as CRISPR-SE and E-CRISP are the best options to be used in all genomes with high accuracy.

## 3 Conclusion

CRISPR systems have been developed for accurate genome editing. Since genomic modifications are permanent (Ding et al., 2018), it is crucial to make precise edits. Most of the tools or methods in CRISPR’s field have been developed to help users design proper gRNA with fewer off-target effects. It is considered that the efficiency of one gRNA may differ among different models and databases. Users must evaluate several gRNAs using multiple models and select the best one for their experiments.

The previous successes of CNN and RNN architectures in bioinformatics motivated other researchers to extend their applications with a DL platform, which we believe is the best solution for predicting off-target effects. DL methods are inexpensive and fast compared to experimental methods. However, their accuracy depends on the amount of available data for a model’s training. Additionally, most of existing methods have three big problems, which means their predictions are not exact. First, they calculate scores based on mismatches to the guide sequence. However, DL-based methods can extract more efficient features hidden in the input data. In other words, DL-based methods can capture features other than gRNA sequence-based features. These features can be utilized and encoded in the input sequence to improve the performance of the existing DL architectures. In addition, most proposed DL-based methods use a one-hot vector representation to encode the input data. (Charlier et al., 2021). The use of newer encoding and embedding methods proposed in the field of DL can enhance the efficiency of existing DL-based methods. Also, the use of gRNA-DNA pair encoding can be helpful. Second, there is a rapid expansion in experimental data in CRISPR research. Most methods cannot scale and improve their performance with this new data. As known, DL-based methods achieve better performance by training on large datasets, but they require a pre-processing step to prepare and aggregate data obtained from diverse sources based on different experimental methods. This step requires enough knowledge about the type of input data, the operation mechanism of CRISPR, and the architecture of the deep neural network. Finally, the most severe issue is that existing DL-based methods still need to be improved in providing sufficient precision for clinical practice usage. NGS-based whole-genome sequencing technologies help to discover almost all off-target sites in the target genome and create a large and more accurate train dataset. As the number of instances in a train dataset increases, the predictions of DL-based methods become closer to experimental observations.
